# Early detection of mesenteric ischemia in critically ill patients following cardiac surgery

**DOI:** 10.1038/s41598-025-10534-9

**Published:** 2025-07-10

**Authors:** Zulfugar T. Taghiyev, Sophia Gunkel, Lili-Marie Beier, Carina Leweling, Kevin M. Sadowski, Borros M. Arneth, Chrysanthi Skevaki, Johannes Kalder, Paula R. Keschenau, Andreas Böning

**Affiliations:** 1 Department of Cardiovascular Surgery, Justus Liebig University, Giessen, Germany; 2 Institute of Laboratory Medicine, German Center of Lung Research, Universities of Giessen and Marburg Lung Center, Phillips University Marburg, Marburg, Germany; 3 Department of Cardiovascular Surgery, University Hospital Giessen, Rudolf-Buchheim-Str. 7, 35392 Giessen, Germany

**Keywords:** Intestinal fatty acid-binding protein (I-FABP), Cardiac surgery, Mesenteric ischemia, Acute gastrointestinal injury (AGI), Diagnostic markers, Predictive markers

## Abstract

Mesenteric ischemia (Me-Is) is a severe complication with high mortality after cardiac surgery. We examined whether intestinal fatty acid binding protein (I-FABP) might serve as a biomarker for early detection of Me-Is following cardiac surgery. Between March 2022 and December 2023, we conducted a prospective observational study of 500 consecutive patients undergoing cardiac surgery with cardiopulmonary bypass. Blood samples were obtained at five time points perioperatively. An immunoassay was used to determine I-FABP levels in 48 individuals at high risk of Me-Is due to lactic acidosis > 4 mmol/L and interleukin (IL)-6 > 600 pg/mL: 6 patients with confirmed Me-Is (cMe-Is), 18 patients with suspected Me-Is (sMe-Is), and 24 patients a in propensity score-matched control group. Of the 48 patients, 62.5% initially had mild gastrointestinal dysfunction (AGI ≤ II), but 80% of them progressed to AGI III or higher, with 25% reaching AGI IV. Baseline serum I-FABP values were similar between the three groups. The six patients with cMe-Is showed significantly elevated serum I-FABP levels from ICU admission to 36 h post-admission, peaking at 207,411.46 pg/mL. ROC analysis identified serum I-FABP as the most accurate predictor of Me-Is, with an AUC of 0.973 at 36 h post-ICU admission (95% CI [0.764–0.997], *p* < 0.001) and an optimal cut-off of > 1421 pg/mL, with 100% sensitivity. In contrast, IL-6 and lactate levels showed no significant differences between groups. Serum I-FABP showed high accuracy in predicting Me-Is after cardiac surgery, outperforming IL-6 and lactate. Our data suggest that I-FABP may have value as a biomarker for early detection of Me-Is after cardiac surgery and help to improve patient management.

**Supplementary Information:**

The online version contains supplementary material available at 10.1038/s41598-025-10534-9.

## Introduction

Extracorporeal circulation in the form of cardio-pulmonary bypass (CPB) is an important contemporary strategy for organ protection during major cardiovascular surgery. Despite protecting the organs from severe ischemia–reperfusion injury, it generates a significant inflammatory response that may lead to organ dysfunction^1–4^. Capillary leak syndrome, an inflammatory reaction after cardiac surgery involving CPB, is associated with increased morbidity and mortality^5^. Microcirculatory alterations in the mesenteric artery during or after CPB contribute to the intestinal hypoperfusion^6^.

Suboptimal microperfusion and ischemia–reperfusion of the intestinal tissue during CPB trigger altered gut permeability and in turn a systemic inflammatory response syndrome^7,8^. The inflammatory reaction with increased interleukin (IL)-6 levels results in microcirculatory dysfunction of the small bowel with a perfusion shift from the muscular toward the mucosal layer^7–9^. This perfusion shift leads to cellular damage that is reflected by increased plasma levels of intestinal fatty acid binding protein (I-FABP) and moderate morphological changes^8,9^. I-FABP is a valuable marker for epithelial injury of the intestine and, as a result, for mesenteric ischemia^7–9^. This cytosolic protein with a molecular weight of 15 kDa is expressed only in mature enterocytes. Because of its low molecular weight and its location at the tips of the intestinal villi, it rapidly infiltrates into the circulation in cases of mucosal damage and is eliminated in the urine^10^.

Only a few clinical studies have prospectively evaluated the high sensitivity and specificity of I-FABP in identification of mesenteric ischemia in patients presenting with acute abdomen after cardiac surgery^11–13^. The diagnostic value of I-FABP was judged to be low in several prior studies that had small study populations^14–16^.

The aim of our study was to prospectively evaluate the serum levels of perioperatively assessed I-FABP and to correlate those with clinical findings in order to improve on a standardized diagnosis. This was a prospective observational trial with 500 registry patients who were undergoing cardiovascular surgery.

## Patients and methods

### Ethics statement

This study was approved by the Institutional Review Board and the Ethics Committee of the Justus Liebig University Giessen (registration number: GI AZ 293/20). The trial was registered on clinicaltrial.gov with the identifier NCT06365827. All participants gave their written informed consent to participate in this prospective registry study. All methods were performed in accordance with the relevant guidelines and regulations.

### Screening and patient cohort

This prospective observational study involved 500 of 929 consecutive surgical patients undergoing open-heart surgery at a single institution from March 2022 to December 2023. Patients with chronic organ dysfunction (e.g. hepatic or renal), confirmed or strongly suspected preoperative infection (e.g. endocarditis), or severe immunodeficiency states were excluded as were cases where informed consent was not obtained.

All patients underwent procedures and medical treatment with close monitoring after the end of surgery until they were discharged from the hospital. The details of the patients were gathered from their electronic records. The research data was organized using the electronic data capture software REDCap at the University Hospital Giessen.

The predictive models described in the present study comply fully with the Transparent Reporting of a multivariable prediction model for Individual Prognosis Or Diagnosis (TRIPOD) guidelines (Supplementary TRIPOD + AI Statement)^17^.

### Collection of samples and laboratory measurements

**Fig. 1:**
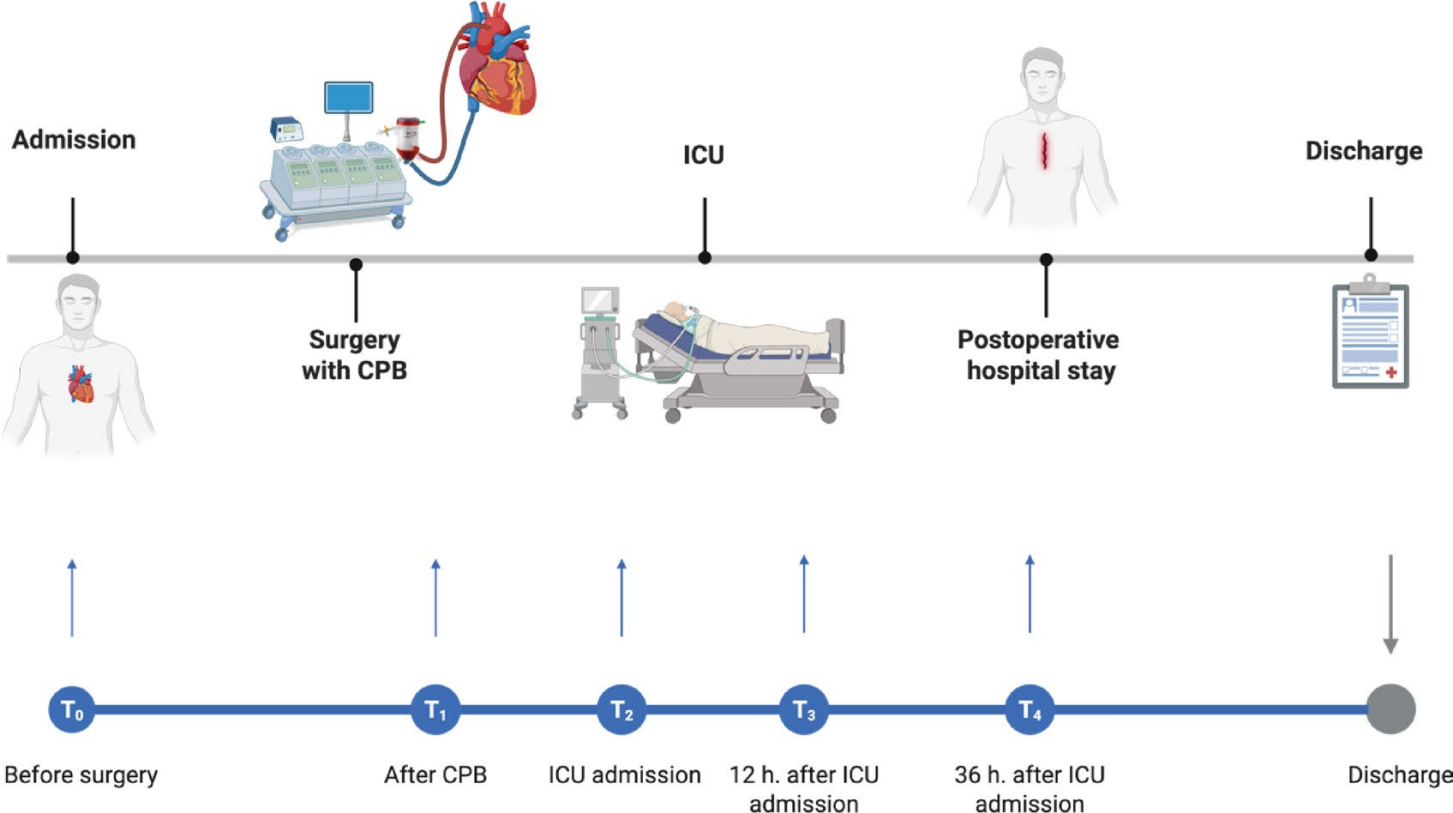
Overview of sampling time points: T_0_ before surgery; T_1_ after protamine administration (after CPB); T_2_ at ICU admission; T_3_ 12 h after ICU admission; T_4_ 36 h after ICU admission.

Blood samples were taken at five time point perioperatively according to the following sampling plan: T_0_—preoperative baseline samples; T_1_—intraoperatively, after the end of CBP and protamine administration; T_2_—postoperatively, at admission to the intensive care unit (ICU); T_3_—12 h after ICU admission; T_4_—36 h after ICU admission. Figure 1 shows an overview of the sampling plan.

In accordance with prior scientific evidence and pathophysiological rationale^18^, we intentionally selected the 36-h time point (T4) as the final sampling interval in our study design to enable a precise characterization of both IL-6 kinetics and the concomitant I-FABP dynamics at this pivotal stage of the postoperative immunoinflammatory course, thereby providing a meaningful window for early prognostic evaluation stratification.

Each blood sample was collected simultaneously with regular perioperative laboratory tests. The samples were centrifuged within 30 min of collection (at 3000 rpm for 10 min at 4˚C), and a 500-µL aliquot was stored at − 80 °C until analysis took place at the Clinical Research Center laboratory at the University of Marburg. I-FABP levels in serum samples were determined using ELISA (high-sensitivity immunoassay kit from Hycult®Biotech (HK406); Hycult Biotechnology B.V., Uden, The Netherlands). The measurement of human I-FABP is feasible within the range of 47 to 3000 pg/ml. In cases where samples exceeded 3000 pg/ml, samples were diluted up to tenfold with a buffer solution in accordance with the manufacturer’s instructions. Levels of lactate and IL‐6 were measured in an in-house accredited clinical laboratory using certified and standardized protocols.

### Target population

To ensure the inclusion of critically ill individuals with a high likelihood of sepsis-associated tissue hypoperfusion, we selected patients presenting with a serum lactate concentration ≥ 4 mmol/L, consistent with internationally accepted definitions of septic shock^19^. This lactate threshold is a well-established surrogate for severe circulatory and metabolic dysfunction and is widely used as a prognostic marker in both clinical guidelines and observational cohorts. Among these patients, the mean interleukin-6 (IL-6) concentration was approximately 600 pg/mL. This degree of cytokinemia is in accordance with previous findings indicating that IL-6 levels in the range of 500–700 pg/mL are strongly associated with adverse outcomes such as organ failure and mortality, particularly in surgical and trauma populations^20–22^. Accordingly, the use of this IL-6 cut-off reflects both the inflammatory phenotype of the enrolled cohort and its prognostic relevance as supported by the literature.

**Fig. 2:**
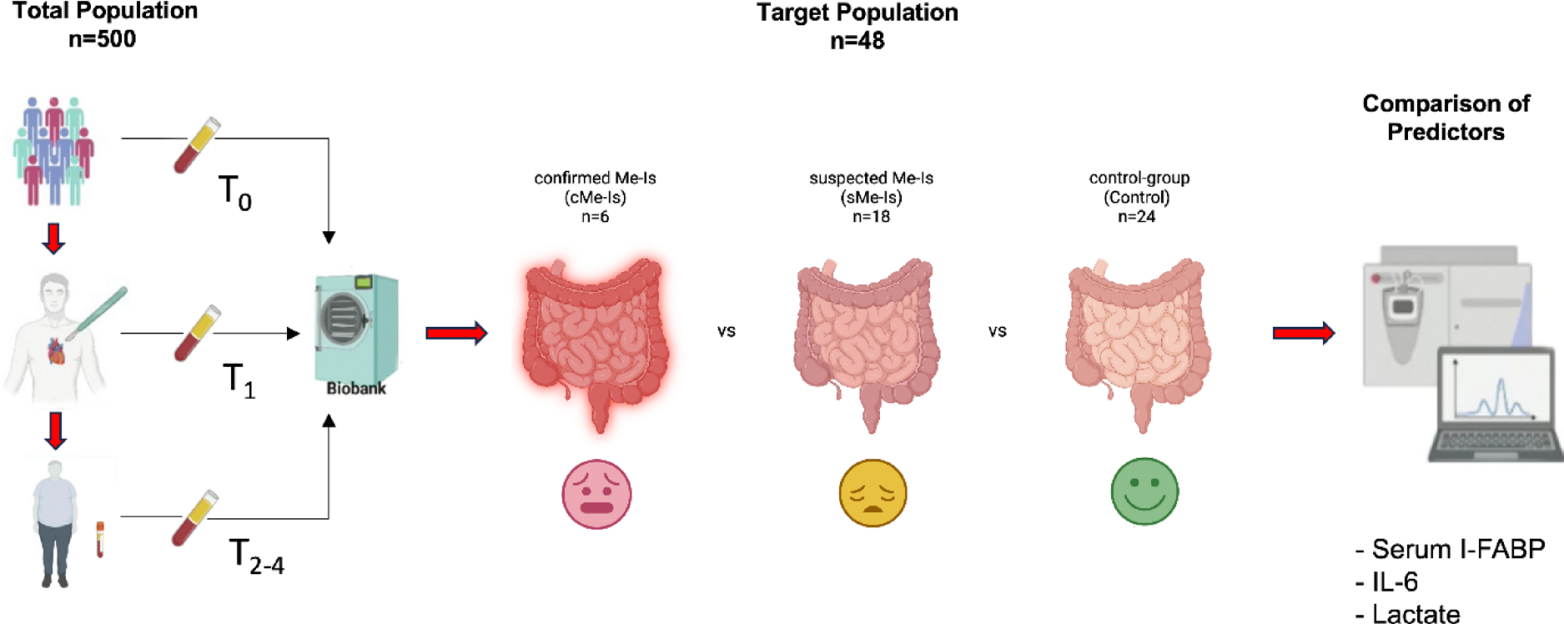
Overview of target population.

Out of 500 consecutive patients who gave informed consent, the study detected six (1.2%) individuals with confirmed mesenteric ischemia and 18 (3.6%) with suspected high risk for mesenteric ischemia. After performing propensity score matching, we included 24 (4.8%) more individuals to serve as a control group. The target population was then divided into three distinct groups: confirmed mesenteric ischemia (cMe-Is), a disease control group defined as suspected mesenteric ischemia (sMe-Is), and a control-group (Fig. 2).

### Statistical analysis

The statistical analyses were conducted using Statistical Package for the Social Sciences (SPSS®) version 26.0 for Mac OS (IBM® Corporation released 2019, Armonk, New York, USA), GraphPad Prism version 8.0.0 for Mac OS (GraphPad Software released 2018, San Diego, California, USA), and NCCS Statistical Analysis and Graphics software version 23.0.2 (released 2023, NCSS, LLC., Kaysville, Utah, USA) following appropriate coding procedures. For continuous variables, the mean ± standard deviation (SD) was reported for normally distributed data, and the median with interquartile range (IQR), defined as Q3–Q1, was reported for non-normally distributed data. Categorical variables were presented as frequencies and percentages. Inter-group disparities across various time points were assessed by one-way variance analysis (ANOVA), with Tukey’s post hoc test being applied in instances of observed differences. The normality of data distribution within each group was evaluated using the Shapiro–Wilk test. Student’s t-test (unpaired) was used for comparison for normally distributed variables, whereas non-normally distributed variables were analyzed using the Mann–Whitney U-test or the Wilcoxon signed-rank test.

Comparisons between different groups were made using Pearson’s chi-squared or Fisher’s exact test to ascertain measurement independence. A standard confidence level of 95% was set, and statistical significance was determined at a *p*-value less than 0.05 (two-tailed). The non-parametric Kruskal–Wallis test was used to examine differences between three independent groups. In multiple comparisons, adjustments were made using the Bonferroni correction method.

Propensity score matching was performed using a 1:4 nearest-neighbor matching algorithm (6 cMe-Is vs. 24 control group) without replacement, applying a caliper width of 0.2 times the pooled standard deviation of the logit-transformed propensity score, in accordance with established recommendations for small sample sizes. Post-matching balance diagnostics demonstrated satisfactory covariate alignment, with pooled standard deviations and caliper widths summarized in Supplementary Table 1. Standardized mean differences (SMDs) were calculated and reported for continuous covariates, including age, BMI, EuroSCORE II, STS score, CPB time, and eGFR, to evaluate the effectiveness of matching. The binary covariate “sex” (male/female) was incorporated into the propensity score model due to its recognized clinical significance; for post-matching balance assessment, binary variables such as sex were evaluated using absolute differences in proportions and chi-square testing.

The main research question was to determine whether elevated serum levels of I-FABP during the perioperative period may be used to distinguish individuals who are at risk of mesenteric ischemia.

## Results

### Demographic data and preoperative morbidity

The mean age of the study population was 66.79 ± 14.14 y with a mean BMI 28.34 ± 5.00 kg/m^2^ (comprising 35 men and 13 women). Further details on demographic parameters for this group are provided in Table 1.

**Table 1:** Characteristics of the study population.

Variable	cMe-Is (n = 6)	sMe-Is (n = 18)	Control (n = 24)
Male, n (%)	5 (83)	12 (66)	18 (75)
BMI, kg/m^2^	26.20 ± 3.93	26.78 ± 4.81	28.97 ± 5.42
Age, y	60.23 ± 29.52	67.07 ± 11.23	67.39 ± 9.53
COPD Gold III-IV, n (%)	0 (0)	1 (5.5)	3 (12.5)
NYHA class III- IV, n (%)	2 (33.3)	13 (72.2)	14 (58.3)
CCS class III-IV, n (%)	2 (33.3)	5 (27.8)	5 (20.8)
Diabetes on insulin, n (%)	0 (0)	3 (16.7)	5 (20.8)
HbA1c, %	6.20 ± 0.67	5.90 ± 0.86	6.14 ± 0.93
Re-do surgery, n (%)	0 (0)	2 (11.1)	5 (20.8)
Ejection fraction, %	52.50 ± 10.61	45.42 ± 16.48	52.65 ± 9.14
Serum creatinine, mg/dl	1.12 ± 0.46	1.11 ± 0.31	1.54 ± 1.48
eGFR, ml/min/1.73 m^2†^	74.63 ± 28.87	69.00 ± 21.08	66.16 ± 27.89
STS-Prom score, predicted mortality, %	6.61 ± 6.56*	3.43 ± 3.42	2.21 ± 2.51
EuroSCORE I	31.90 ± 20.90*	12.74 ± 11.08	10.08 ± 9.61
EuroSCORE II	12.33 ± 11.16	9.14 ± 8.62	6.44 ± 6.29

BMI, Body mass index; COPD, Chronic obstructive pulmonary disease; NYHA, New York Heart Association; CCS, Canadian Cardiovascular Society; eGFR, Estimated glomerular filtration rate; STS, Society of Thoracic Surgeons.

^†^Cockcroft-Gault Equation^23^.

*p*-values for comparison: *, < 0.05; **, < 0.01; ***, < 0.001.

The Acute Gastrointestinal Injury (AGI) score^24^ was used to assess gastrointestinal dysfunction upon ICU admission. Approximately 62.5% (30/48) of patients had an AGI score of grade II or less during their stay in the ICU. Out of these patients, around 80% (24/30) progressed to grade III or higher in the following days. Subsequently, 25% of patients (6/24) had advanced AGI grade IV.

For the 48-patient cohort, the average ICU duration was 5 days (ranging from 1.9 to 11.5 days) and the in-hospital mortality rate was 21%. In the ICU, dialysis was applied to 14 patients. A mechanical assist device was implanted in 17% of the total target population. The median [Q1–Q3] duration of invasive mechanical ventilation across the groups was 52.0 [26.3–203.0] in cMe-Is group vs. 36.7 [13.9–85.1] in the sMe-IS group vs. 24.7 [15.4–41.5] the in control group. Hemofiltration was administered to 14 patients. A need for mechanical circulatory support was registered in 23% of the target population (Table 2).

**Table 2:** Perioperative characteristics of the groups.

Variable	cMe-Is (n = 6)	sMe-Is (n = 18)	Control (n = 24)
Intraoperative characteristics			
Aortic surgery, n (%)	1 (16.7)	1 (5.6)	2 (8.3)
CABG surgery, n (%)	1 (16.7)	4 (22.2)	9 (37.5)
Valve surgery, n (%)	2 (33.3)	2 (11.1)	4 (16.7)
Combined surgery, n (%)	2 (33.3)	11 (61.1)	9 (37.5)
CPB time, hh: mm	2:04 ± 0:43	2:21 ± 0:39	2:19 ± 1:19
Cross-clamp time, hh: mm	1:06 ± 0:16	1:31 ± 0:38	1:32 ± 0:54
Reperfusion time, hh: mm	0:42 ± 0:29	0:32 ± 0:16	0:33 ± 0:30
Postoperative characteristics			
Need for assist devices, n (%)	3 (50.0)*	6 (33.3)	2 (8.3)
ICU stay, days	2.9 [1.0–5.7]	11.7 [5.2–28.0]**	2.8 [1.2–6.9]
Mean invasive ventilation duration, h	101.2 ± 117.2	75.9 ± 100.4	40.49 ± 80.2
Dialysis, n (%)	1 (16.7)	8 (44.4)	5 (20.8)
Re-thoracotomy, n (%)	2 (11.1)	6 (33.3)	2 (8.3)
APACHE II score	22.0 [16.0–25.0]	17.0 [10.0–25.5]	22.0 [18.0–24.5]
SOFA score	9.0 [6.0–10.0]	5 [4.0–9.8]	7 [5.5–9.0]
SAPS II	37.0 [32.5–41.5]	42.5 [29.0–56.0]	35 [32.5–41.5]
TISS 10	28.0 [22.0–31.0]	19.0 [11.3–28.3]	22 [18.0–29.0]
Norepinephrine support, µg/kg	395.8 ± 382.9	723.9 ± 778.5	394.9 ± 896.5
In-hospital mortality, n (%)	5 (83.3)***	4 (22.2)	1 (4.2)
Stroke, n (%)	0 (0)	2 (11.1)	1 (4.2)
Hospital stay, days	7.0 [5.5–11.0]	23.5 [15.5–37.5]***	13.0 [10.7–14.0]

eGFR, Estimated glomerular filtration rate; CABG, Coronary Artery Bypass Graft surgery; CPB, Cardiopulmonary bypass; ICU, Intensive care unit; APACHE II, Acute Physiology and Chronic Health Evaluation II; SOFA, Sequential Organ Failure Assessment.

*p*-values for comparison: *, < 0.05; **, < 0.01; ***, < 0.001.

### I-FABP levels and intervals to mesenteric ischemia

A total of 18 patients in the sMe-Is group presented with clinical symptoms of bowel ischemia; however, no endoscopic evidence of ischemia was observed (in eight cases), nor was ischemia detected on CT examinations (in ten cases) or during laparoscopic surgeries (in two cases).

In the cMe-IS group, one individual was diagnosed with NOMI (*non-occlusive mesenteric ischemia*) within 12 h after ICU admission and was treated with prostaglandin perfusion. The remaining five patients of the cMe-Is group underwent laparotomy because they showed symptoms indicating widespread peritonitis 2 or 3 days after being admitted to the ICU. Mesenteric ischemia was confirmed intraoperatively and by histological examination in all five cases.

**Fig. 3:**
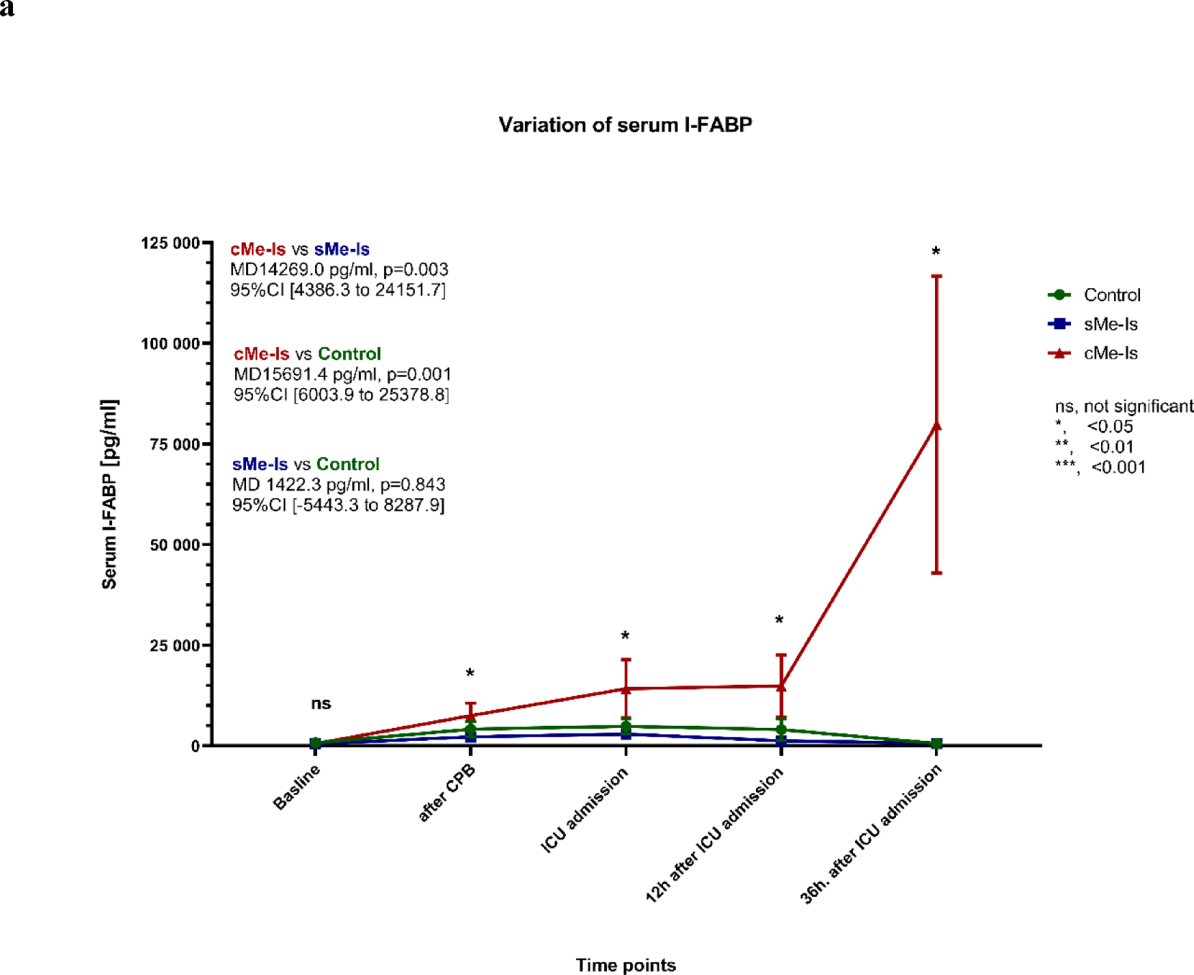

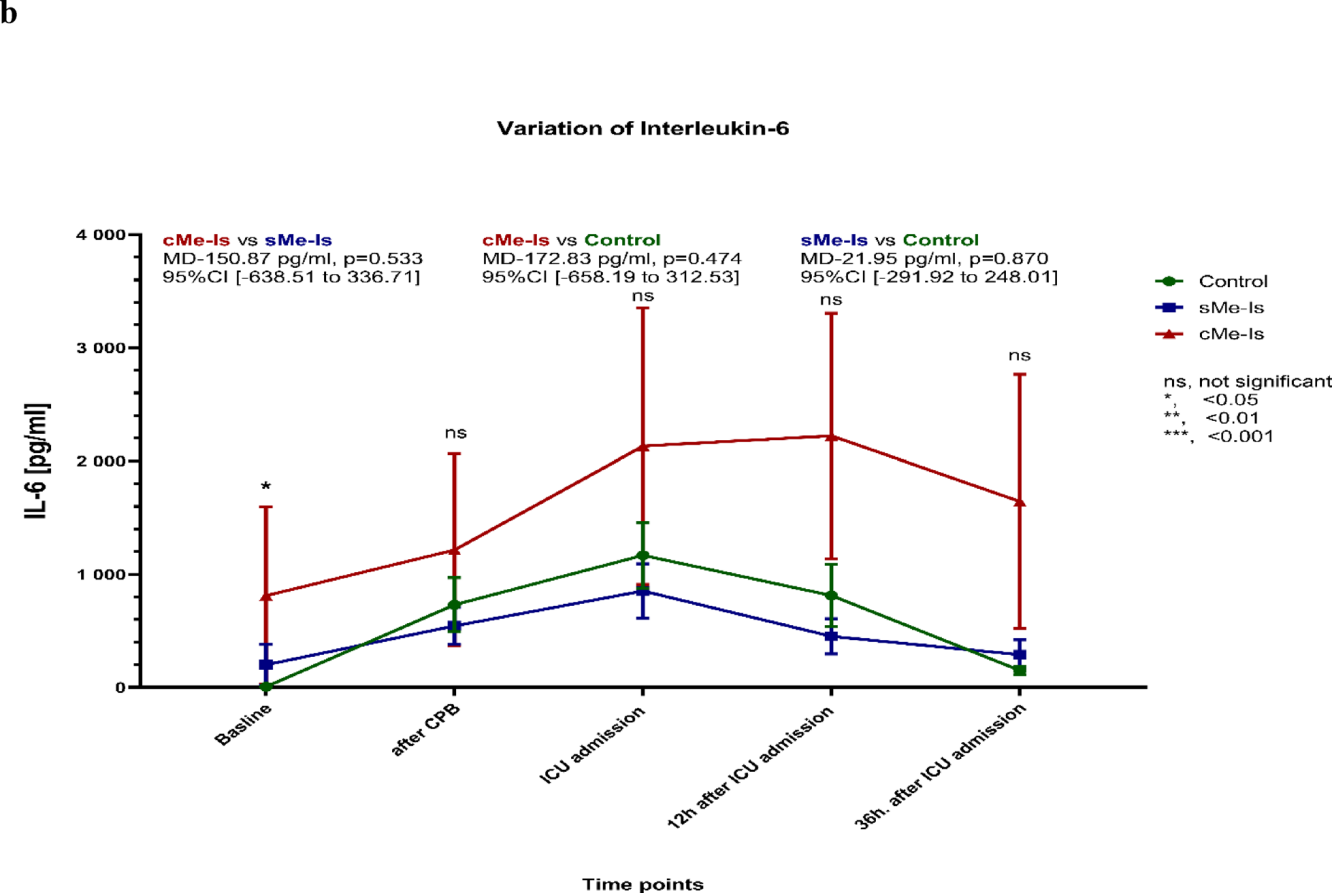

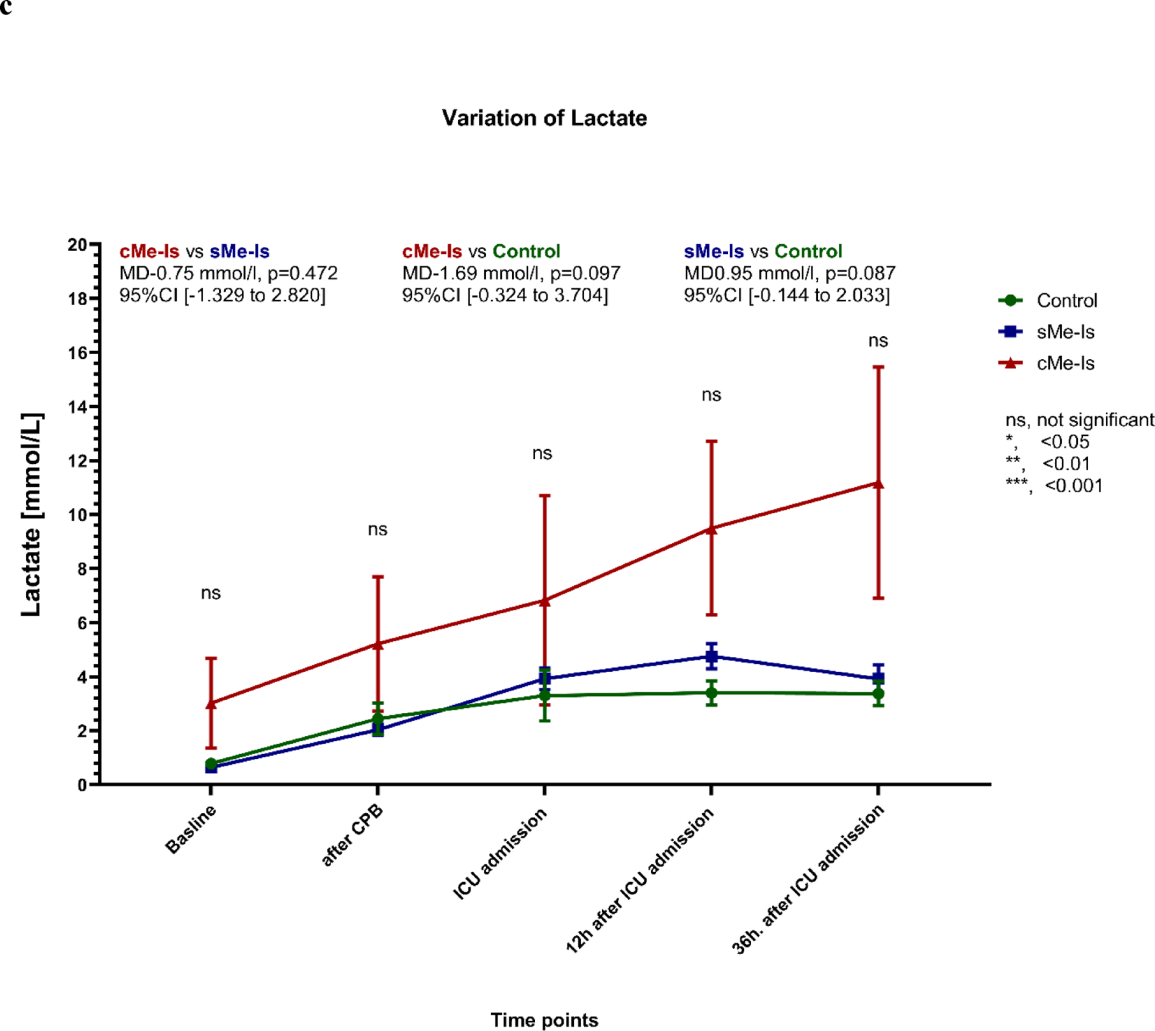
Time course of the mean (± SEM) levels of serum (**a**) I-FABP, (**b**) IL-6 and (**c**) lactate for the three groups. The mean differences (MD) in I-FABP levels between the groups were significant from ICU admission onwards. *p*-values for comparison: ns, nonsignificant; *, < 0.05; **, < 0.01; ***, < 0.001. CPB, Cardio-pulmonary bypass; ICU, Intensive care unit; sMe-Is, Suspected mesenteric ischemia; cMe-Is, Confirmed mesenteric ischemia.

The median [Q1-Q3] baseline levels of serum I-FABP were 541.16 [287.05–860.68] pg/mL (mean 625.72 ± 487.47 pg/mL), with no notable differences between the three groups. Patients in the disease and control groups exhibited comparable levels of I-FABP at each measured time point (*p* = 0.965; Fig. 3a). The six patients with bowel ischemia had significantly increased I-FABP levels 36 h after ICU admission, with a maximum value of 207,411.46 pg/ml. In the perioperative period, there were no significant differences in IL-6 or lactate levels between the groups (Fig. 3b, c).

### Prediction of mesenteric ischemia

To evaluate whether serum I-FABP can serve as a marker for predicting of mesenteric ischemia, we utilized receiver operating characteristics (ROC) analysis of I-FABP levels at five different time points to identify the time with the highest predictive accuracy. Table 3 shows that I-FABP had the highest diagnostic value 12 h after ICU admission (T3), with an AUC of 82.4% (*p* = 0.013, 95%CI [0.272 to 0.968].

**Table 3:** Comparison of the accuracy of serum I-FABP across five time points.

Time points	AUC	*p* (1-sided)	95% CI	
			Lower	Upper
Baseline	0.527	0.408	0.264	0.717
After CPB	0.770	0.040	0.272	0.943
After ICU admission	0.775	0.033	0.292	0.943
12 h after ICU admission	0.824	0.013	0.272	0.968
36 h after ICU admission	0.779	0.030	0.295	0.945

CPB, Cardiopulmonary bypass; ICU, Intensive care unit; CI, Confidence interval.

**Fig. 4:**
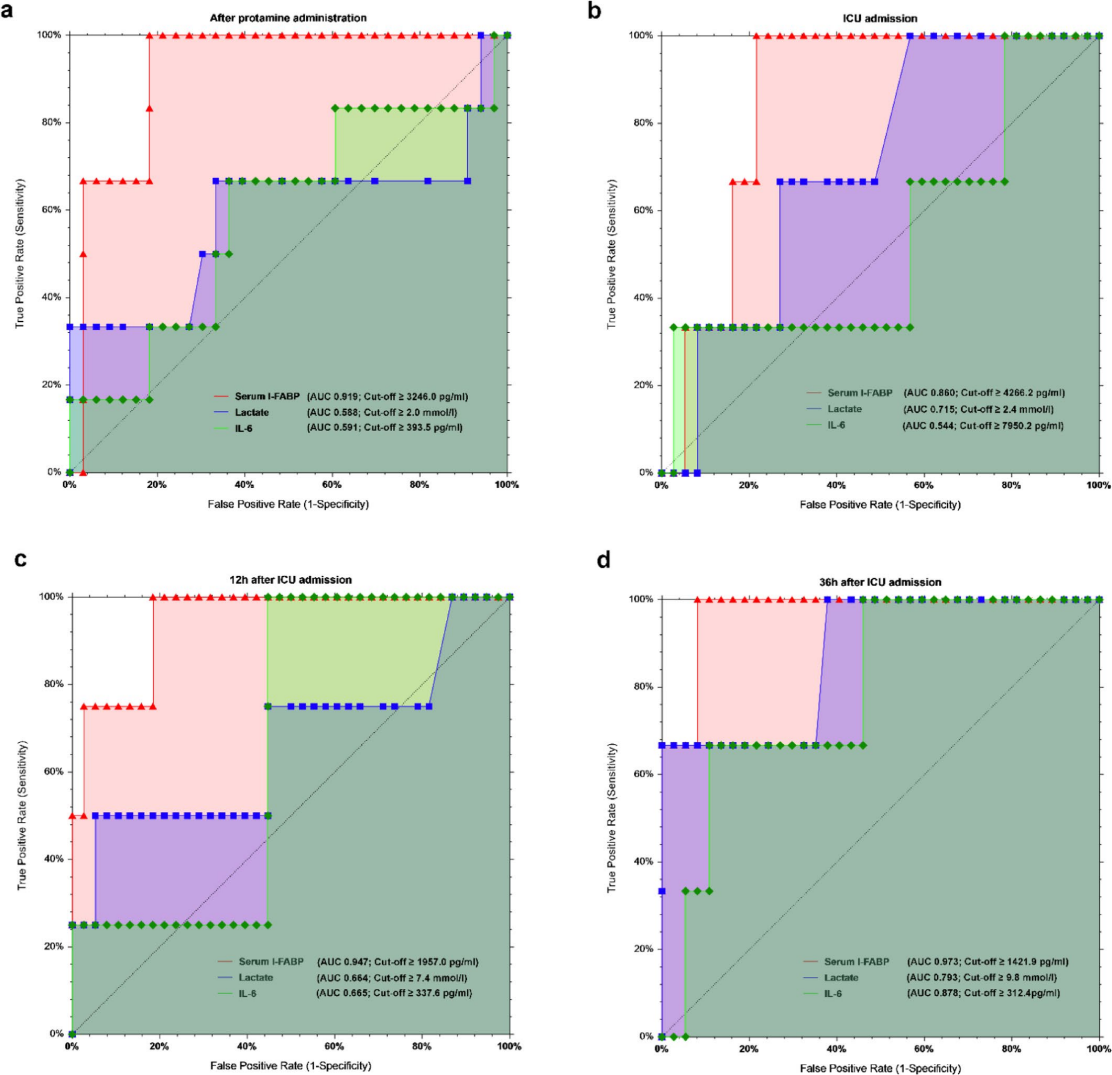
ROC analyses for prediction of mesenteric ischemia for three parameters at different time points (**a**–**d**). ICU, Intensive care unit; I-FABP, Intestinal fatty acid-binding protein in serum; IL-6, Interleukin 6.

Sensitivity, specificity, and AUC were calculated and compared for serum I-FABP, IL-6, and lactate at four perioperative time points (Fig. 4). In addition to the previously described findings, Table 4 provides a comprehensive summary of the diagnostic performance measures for each biomarker (I-FABP, IL-6, lactate) across all time points. Specifically, the ROC analysis revealed that after protamine administration (T₁), I-FABP demonstrated an AUC of 0.919 (95% CI [0.753–0.975], *p* = 0.000) with 100% sensitivity, 82% specificity, and 84.62% accuracy at a cut-off ≥ 3246.0 pg/mL. At ICU admission (T_2_), I-FABP showed an AUC of 0.860 (95% CI [0.659–0.946], *p* = 0.000), 100% sensitivity, 79.0% specificity, and 80.49% accuracy (cut-off ≥ 4266.2 pg/mL). At 12 h after ICU admission (T₃), I-FABP reached an AUC of 0.947 (95% CI [0.707–0.992], *p* = 0.000), with 100% sensitivity, 81.6% specificity, and 83.30% accuracy (cut-off ≥ 1957.0 pg/mL). Finally, at 36 h after ICU admission (T₄), I-FABP achieved the highest AUC of 0.973 (95% CI [0.764–0.997], *p* = 0.000), with 100% sensitivity, 91.9% specificity, and 92.50% accuracy (cut-off ≥ 1421.0 pg/mL).

**Table 4:** Diagnostic accuracy of I-FABP, IL-6, and lactate for predicting mesenteric ischemia after cardiac surgery.

Time points	Predictor	AUC	Cut-off	95% CI	*p* (1-sided)	Sensivity	Specificity	Accuracy
After protamine administration (T_1_)	I-FABP	0.919	≥ 3246.0 pg/ml	0.753 to 0.975	0.000	100	82	84.62
	IL-6	0.591	≥ 393.5 pg/ml	− 0.233 to 0.808	0.268	66.7	63.6	64.10
	Lactate	0.588	≥ 2.0 mmol/l	− 0.149 to 0.834	0.307	66.7	66.7	66.70
ICU admission (T_2_)	I-FABP	0.860	≥ 4266.2 pg/ml	0.659 to 0.946	0.000	100	79.0	80.49
	IL-6	0.544	≥ 7950.2 pg/ml	− 0.031 to 0.848	0.424	33.3	97.4	92.68
	Lactate	0.715	≥ 2.4 mmol/l	− 0.336 to 0.895	0.058	100	44.8	48.78
12 h after ICU admission (T_3_)	I-FABP	0.947	≥ 1957.0 pg/ml	0.707 to 0.992	0.000	100	81.6	83.30
	IL-6	0.665	≥ 337.6 pg/ml	− 0.103 to 0.905	0.204	100	55.26	59.52
	Lactate	0.664	≥ 7.4 mmol/l	− 0.339 to 0.848	0.099	50.0	94.7	90.50
36 h after ICU admission (T_4_)	I-FABP	0.973	≥ 1421.0 pg/ml	0.764 to 0.997	0.000	100	91.9	92.50
	IL-6	0.878	≥ 312.4 pg/ml	0.292 to 0.985	0.001	67.0	89.2	87.50
	Lactate	0.793	≥ 9.8 mmol/l	0.351 to 0.946	0.015	66.7	100	97.50

I-FABP, Intestinal fatty acid-binding protein; IL-6, Interleukin-6; AUC, Area under the curve; CI, Confidence interval.

For IL-6, the respective AUCs were 0.591 (T_1_), 0.544 (T_2_), 0.665 (T_3_), and 0.878 (T_4_), with corresponding cut-offs and diagnostic parameters as detailed in Table 4. For lactate, the AUCs were 0.588 (T_1_), 0.715 (T_2_), 0.664 (T_3_), and 0.793 (T_4_), also with full diagnostic metrics reported (Table 4).

All six patients with mesenteric ischemia had significantly high serum I-FABP levels from the time of admission to the ICU. Assuming a cut-off level at 1421 pg/mL, assay sensitivity was 100%.

## Discussion

I-FABP is a promising serum marker for diagnosing intestinal ischemia, a rare but serious complication of cardiac surgery. It is released into the circulation during the acute phase of mesenteric ischemia, making it a useful diagnostic tool. While I-FABP is not traditionally associated with cardiac surgery, its role as a biomarker for intestinal tissue injury suggests potential utility in this context. The present research focused primarily on comparing serum I-FABP and established biomarkers for predicting mesenteric ischemia after cardiac surgery. Two control groups were defined: disease controls, defined as those having clinical signs of mesenteric ischemia, and a control group with no signs of ischemia. In our study, the sensitivity, specificity, and area under the curve (AUC) for serum I-FABP were highest at any time point.

Several other groups have conducted studies of the utility of I-FABP. Van der Voort et al. investigated the effectiveness of I-FABP in detecting mesenteric ischemia among 44 ICU patients who were suspected of having the condition^14^. Their study found that median I-FABP levels were 2872 pg/mL in patients who developed ischemia and 1020 pg/mL in those who did not. However, the difference between the two groups was not statistically significant due to the wide variation in I-FABP levels. One possible explanation for the consistently high levels in the two groups is the high prevalence of mesenteric hypoperfusion among critically ill patients. Another important factor to consider is that the study did not report the time interval between surgery and I-FABP testing. This is particularly relevant, because in ICU patients the exact onset of mesenteric ischemia is often unclear; clinical symptoms such as acute abdominal pain can be masked by the patient’s overall condition. The timing of the I-FABP measurement relative to the hypoperfusion event could significantly affect its diagnostic reliability. If the test is performed too late, it may yield false-negative results, whereas testing too early could lead to false positives.

To account for this, our study measured serum I-FABP levels at two different time points during the ICU stay. By conducting four measurements—starting immediately after cardiopulmonary bypass (CPB) and continuing throughout the ICU stay—it may be possible to reliably confirm or rule out future mesenteric ischemia. These findings suggest that repeated I-FABP testing in high-risk scenarios, such as major cardiac surgery or cardiogenic shock, may provide a more accurate picture of intestinal injury and its progression. However, for this approach to be widely used in clinical practice, a rapid and accessible laboratory test would be necessary.

Similarly, a study by Ludewig et al. found that in ICU patients, a single I-FABP measurement taken at the time when mesenteric ischemia was clinically suspected was not a reliable way to confirm or rule out the condition^16^. The test only demonstrated high diagnostic accuracy when performed between 12 and 48 h after the onset of ischemia. This suggests that I-FABP is most useful for perioperative monitoring rather than for early detection. Future studies should further examine how the timing of the test affects its reliability in diagnosing mesenteric ischemia.

In our study, serum I-FABP levels were generally low and showed no significant differences between the two control groups. In contrast, all six patients diagnosed with mesenteric ischemia had I-FABP levels above the cut-off of 1421 pg/mL. Our findings suggest that serum I-FABP is a highly promising diagnostic marker for mesenteric ischemia with a sensitivity of 100%, which is far superior to conventional biochemical markers.

Our study additionally confirmed an important characteristic that had been previously observed in experimental research in a rat model. Kanda et al. have shown that a temporary blockage of the superior mesenteric artery, which causes reversible damage to the intestinal mucosa, leads to an immediate rise in serum I-FABP levels^12^. Consistent with these findings, one patient in our study who had NOMI also exhibited high I-FABP levels upon ICU admission. However, after receiving prostavasin treatment targeting the superior mesenteric artery, the levels dropped rapidly. Based on this, we believe the early elevation of I-FABP serves as a reliable indicator of bowel ischemia.

A temporary spike in I-FABP, however, does not necessarily indicate the progression to full-scale bowel necrosis. Instead, it may simply reflect transient mesenteric hypoperfusion, a condition that can be reversed if blood flow is restored in time. Supporting this idea, a study by Vermeulen Windsant et al. found that patients undergoing open aortic surgery had serum I-FABP levels as high as 2298 ± 490 pg/mL without developing any mesenteric complications^15^. Their I-FABP levels returned to normal within a day after surgery.

In addition to the factors already discussed, our previous analyses have shown that elevated I-FABP levels can be influenced by clinical variables such as reduced renal function (e.g., decreased eGFR, elevated creatinine), multiple surgical interventions, longer cross-clamp durations, and higher APACHE II scores, all of which contribute to increased intestinal stress and impaired biomarker clearance^18^. Notably, while these factors can elevate I-FABP independent of acute ischemia, they do not negate its utility as a biomarker, as I-FABP remained an independent predictor of event even after adjusting for these confounders. Furthermore, the diagnostic value of I-FABP may be enhanced by incorporating serial measurements over time, rather than relying on a single time point, and by integrating these data with other clinical indicators such as IL-6, lactate, and clinic presentation to achieve a more precise and practical assessment of intestinal ischemia in postoperative cardiac patients.

Mesenteric ischemia in ICU patients is a serious but often overlooked condition, especially after cardiovascular surgery. Its symptoms are usually vague, which makes it difficult to diagnose. Because of this, the actual number of cases is likely higher than reported^25^. In its subacute form, it can go undetected if the patient’s condition appears stable, yet it may still negatively affect recovery. Several factors increase the risk of mesenteric ischemia, including multiple underlying health conditions, the use of vasopressors, and elevated inflammatory markers. Interestingly, these same factors also seem to influence blood levels of I-FABP.

In the context of cardiac surgery, routine I-FABP measurement is not yet available. The establishment of a rapid test could help identify patients with suspected intestinal ischemia at an early stage. Its diagnostic value is strongest after 36 h; however, earlier time points show higher sensitivity and are more reliable than other control parameters such as IL-6 and lactate.

### Study limitations

This study has some limitations, since it relies on a non-randomized analysis of prospectively gathered registry data from a relatively small group of patients who were all treated at a single center, which restricts the statistical power and generalizability of the findings in this subgroup. The small size of the verified mesenteric ischemia subgroup (n = 6) further limits statistical robustness. Additionally, there is a potential for selection bias within the control population, which must be considered when interpreting the results. Moreover, the I-FABP cut-off values identified in this study require external validation in independent patient cohorts before broad clinical application. Even though propensity score matching was used, there were still notable differences in preoperative characteristics among the three treatment groups.

### Summary and conclusions

This study underscores the potential of I-FABP as a valuable biomarker for predicting bowel ischemia in patients undergoing heart surgery. By identifying those at higher risk in the early postoperative phase, I-FABP could help guide the need for further diagnostic tests like contrast CT or angiography as well as medical interventions such as arterial infusion therapy. To improve patient outcomes, it may be crucial to minimize damage to intestinal cells during surgery and quickly detect signs of intestinal ischemia. Early identification of patients at risk can lead to faster treatment and better survival rates. Looking ahead, larger multicenter studies are needed to confirm these findings and explore whether I-FABP could also serve as a diagnostic target to reduce the risk of postoperative mesenteric ischemia.

## Electronic supplementary material

Below is the link to the electronic supplementary material.

Supplementary Material 1

Supplementary Material 2

Supplementary Material 3

## Data Availability

The article’s data will be shared upon reasonable request to the corresponding author.

## Abbreviations

ANOVA: Analysis of variance
BMI: Body mass index
CI: Confidence interval
CPB: Cardiopulmonary bypass
eGFR: Estimated glomerular filtration rate
ICU: Intensive care unit
I-FABP: Intestinal fatty acid binding protein
IL-6: Interleukin 6
OR: Odds ratio
SD: Standard deviation
SEM: Standard error of the mean
STS Prom: Society of Thoracic Surgeons Predicted Risk of Mortality

## References

[1] CS Ng S Wan AA Arifi 2006 Inflammatory response to pulmonary ischemia-reperfusion injury Surg. Today 36 205 214 10.1007/s00595-005-3124-2 16493527 10.1007/s00595-005-3124-2

[2] WH Huang JF Lee D Wang 2010 Postischemia myocardial injury in coronary artery bypass patients; PP6 Transplant. Proc. 42 725 728 10.1016/j.transproceed.2010.02.067 20430157 10.1016/j.transproceed.2010.02.067

[3] O Liangos S Domhan C Schwager 2010 Whole blood transcriptomics in cardiac surgery identifies a gene regulatory network connecting ischemia reperfusion with systemic inflammation PLoS ONE 5 e13658 10.1371/journal.pone.0013658 21048961 10.1371/journal.pone.0013658 PMC2965092

[4] R Hall 2013 Identification of inflammatory mediators and their modulation by strategies for the management of the systemic inflammatory response during cardiac surgery J. Cardiothorac. Vasc. Anesth. 10.1053/j.jvca.2012.09.013 23276596 10.1053/j.jvca.2012.09.013

[5] R Kubicki J Grohmann M Siepe 2013 Early prediction of capillary leak syndrome in infants after cardiopulmonary bypass Eur. J. Cardiothorac. Surg. 44 2 275 281 10.1093/ejcts/ezt028 23389476 10.1093/ejcts/ezt028

[6] A Guillaume S Pili-Floury S Chocron 2017 Ami after cardiac surgery in critically ill patients Shock 10.1097/SHK.0000000000000720 28195969 10.1097/SHK.0000000000000720

[7] PR Keschenau S Ribbe M Tamm SJ Hanssen R Tolba MJ Jacobs J Kalder 2016 Extracorporeal circulation increases proliferation in the intestinal mucosa in a large animal model J. Vasc. Surg. 64 4 1121 1133 10.1016/j.jvs.2015.05.043 26190050 10.1016/j.jvs.2015.05.043

[8] J Kalder PR Keschenau SJ Hanssen A Greiner IC Vermeulen Windsant LN Kennes 2012 The impact of selective visceral perfusion on intestinal macrohemodynamics and microhemodynamics in a porcine model of thoracic aortic cross-clamping J. Vasc. Surg. 56 149 158 22494690 10.1016/j.jvs.2011.11.126

[9] J Kalder D Ajah PR Keschenau LN Kennes R Tolba M Kokozidou MJ Jacobs TA Koeppel 2015 Microcirculatory perfusion shift in the gut wall layers induced by extracorporeal circulation J. Vasc. Surg. 61 2 497 503 10.1016/j.jvs.2013.10.070 24275079 10.1016/j.jvs.2013.10.070

[10] NJ Evennett MS Petrov A Mittal JA Windsor 2009 Systematic review and pooled estimates for the diagnostic accuracy of serological markers for intestinal ischemia World J. Surg. 33 7 1374 1383 10.1007/s00268-009-0074-7 19424744 10.1007/s00268-009-0074-7

[11] T Kanda A Tsukahara K Ueki 2011 Diagnosis of ischemic small bowel disease by measurement of serum intestinal fatty acid-binding protein in patients with acute abdomen: A multicenter, observer-blinded validation study J. Gastroenterol. 46 4 492 500 10.1007/s00535-011-0373-2 21298292 10.1007/s00535-011-0373-2

[12] JM Lieberman J Sacchettini C Marks WH Marks 1997 Human intestinal fatty acid binding protein: Report of an assay with studies in normal volunteers and intestinal ischemia Surgery. 121 3 335 342 10.1016/s0039-6060(97)90363-9 9068676 10.1016/s0039-6060(97)90363-9

[13] B Relja M Szermutzky D Henrich M Maier JJ Haan de T Lubbers WA Buurman I Marzi 2010 Intestinal-FABP and liver-FABP: Novel markers for severe abdominal injury Acad. Emerg. Med. 17 7 729 735 10.1111/j.1553-2712.2010.00792.x 20653587 10.1111/j.1553-2712.2010.00792.x

[14] PH Voort van der B Westra JP Wester RJ Bosman I Stijn van IA Haagen FJ Loupatty S Rijkenberg 2014 Can serum L-lactate, D-lactate, creatine kinase and I-FABP be used as diagnostic markers in critically ill patients suspected for bowel ischemia BMC Anesthesiol. 14 111 10.1186/1471-2253-14-111 25844063 10.1186/1471-2253-14-111 PMC4384375

[15] IC Vermeulen Windsant FA Hellenthal JP Derikx MH Prins WA Buurman MJ Jacobs GW Schurink 2012 Circulating intestinal fatty acid-binding protein as an early marker of intestinal necrosis after aortic surgery: A prospective observational cohort study Ann. Surg. 255 4 796 803 10.1097/SLA.0b013e31824b1e16 22367448 10.1097/SLA.0b013e31824b1e16

[16] S Ludewig R Jarbouh M Ardelt H Mothes F Rauchfuß R Fahrner J Zanow U Settmacher 2017 Bowel ischemia in ICU patients: Diagnostic value of I-FABP depends on the interval to the triggering event Gastroenterol. Res. Pract. 2017 2795176 10.1155/2017/2795176 28630622 10.1155/2017/2795176 PMC5467337

[17] GS Collins JB Reitsma DG Altman 2015 Transparent reporting of a multivariable prediction model for individual prognosis or diagnosis (TRIPOD): The TRIPOD Statement BMC Med. 13 1 10.1186/s12916-014-0241-z 25563062 10.1186/s12916-014-0241-z PMC4284921

[18] Taghiyev, Z. T., Leweling, C., Beier L.-M. et al. Intestinal Damage Marker as a Potential Predictor of Early Mortality after Cardiac Surgery, 18 December 2024, PREPRINT (Version 1) available at Research Square. 10.21203/rs.3.rs-5382002/v1.

[19] L Evans A Rhodes W Alhazzani 2021 Surviving sepsis campaign: international guidelines for management of sepsis and septic shock 2021 Intensive Care Med. 47 11 1181 1247 10.1007/s00134-021-06506-y 34599691 10.1007/s00134-021-06506-y PMC8486643

[20] M Frink M Griensven van P Kobbe 2009 IL-6 predicts organ dysfunction and mortality in patients with multiple injuries Scand. J. Trauma Resusc. Emerg. Med. 17 49 10.1186/1757-7241-17-49 19781105 10.1186/1757-7241-17-49 PMC2763001

[21] D Geisler N Arleth J Grabenwöger 2023 Impact of CytoSorb® on interleukin-6 in cardiac surgery Front. Cardiovasc. Med. 10 1166093 10.3389/fcvm.2023.1166093 37711559 10.3389/fcvm.2023.1166093 PMC10498300

[22] NI Shapiro MD Howell D Talmor 2005 Serum lactate as a predictor of mortality in emergency department patients with infection Ann. Emerg. Med. 45 5 524 528 10.1016/j.annemergmed.2004.12.006 15855951 10.1016/j.annemergmed.2004.12.006

[23] DW Cockcroft MH Gault 1976 Prediction of creatinine clearance from serum creatinine Nephron 16 1 31 41 1244564 10.1159/000180580

[24] A Reintam Blaser ML Malbrain J Starkopf 2012 Gastrointestinal function in intensive care patients: Terminology, definitions and management. Recommendations of the ESICM Working Group on Abdominal Problems Intensive Care Med. 38 3 384 94 22310869 10.1007/s00134-011-2459-y PMC3286505

[25] RM Gore KH Thakrar UK Mehta J Berlin V Yaghmai GM Newmark 2008 Imaging in intestinal ischemic disorders Clin. Gastroenterol. Hepatol. 6 8 849 858 10.1016/j.cgh.2008.05.007 18674733 10.1016/j.cgh.2008.05.007

