# Supplementary material for: Early detection of mesenteric ischemia in critically ill patients following cardiac surgery
Source: Sci Rep. 2025 Jul 10;15:24854. doi: 10.1038/s41598-025-10534-9 (PMC12246057; doi:10.1038/s41598-025-10534-9)
Supplement: Supplementary file 2 — Supplementary Material 2 [file 41598_2025_10534_MOESM2_ESM.docx]

**Supplementary Table 1.** Overview of matching variables used in the propensity score matching process, showing mean ± SD for the cMe-Is, sMe-Is, and control groups. The pooled standard deviation and caliper width (0.2 × pooled SD) were calculated for the primary matching comparison (cMe-Is vs. control) to guide balance diagnostics.

| **Variable** | **cMe-Is, Mean (SD), n=6** | **sMe-Is Mean (SD), n=18** | **Control Mean (SD), n=24** | **Pooled SD** | **Caliper Width** | **SMD** | **P-value** |
| --- | --- | --- | --- | --- | --- | --- | --- |
| **Age (years)** | 60.23 ± 29.52 | 67.07 ± 11.23 | 67.39 ± 9.53 | 21.93 | 4.39 | -0.33 | 0.582 |
| **BMI (kg/m²)** | 26.2 ± 3.93 | 26.78 ± 4.81 | 28.97 ± 5.42 | 4.73 | 0.95 | -0.59 | 0.185 |
| **Sex (male %)** | 83.0 | 66.0 | 75.0 | 0.4 | 0.08 | 0.21 | 1.00 |
| **EuroSCORE II (%)** | 12.33 ± 11.16 | 9.14 ± 8.62 | 6.44 ± 6.29 | 9.06 | 1.81 | 0.65 | 0.261 |
| **STS Score (%)** | 6.61 ± 6.56 | 3.43 ± 3.42 | 2.21 ± 2.51 | 4.97 | 0.99 | 0.89 | 0.163 |
| **CPB time (min)** | 124.0 ± 43.0 | 141.0 ± 39.0 | 139.0 ± 79.0 | 63.6 | 12.72 | -0.24 | 0.539 |
| **eGFR (ml/min/1.73 m²)** | 74.63 ± 28.87 | 69.0 ± 21.08 | 66.16 ± 27.89 | 28.38 | 5.68 | 0.3 | 0.537 |

Abbreviations: eGFR= estimated glomerular filtration rate; BMI= body mass index; CPB=cardiopulmonary bypass; SMD= standardized mean difference; STS= Society of Thoracic Surgeons
